# Norepinephrine enhances the LPS-induced expression of COX-2 and secretion of PGE_2 _in primary rat microglia

**DOI:** 10.1186/1742-2094-7-2

**Published:** 2010-01-11

**Authors:** Johannes CM Schlachetzki, Bernd L Fiebich, Elisabeth Haake, Antonio CP de Oliveira, Eduardo Candelario-Jalil, Michael T Heneka, Michael Hüll

**Affiliations:** 1 Department of Psychiatry and Psychotherapy, University Hospital of Freiburg, Freiburg, Germany; 2 Division of Molecular Neurology, University of Erlangen, Erlangen, Germany; 3 Department of Neurology, University of New Mexico, Albuquerque, NM, USA; 4 Department of Neurology, Clinical Neurosciences, University of Bonn Medical Center, Bonn, Germany

## Abstract

**Background:**

Recent studies suggest an important role for neurotransmitters as modulators of inflammation. Neuroinflammatory mediators such as cytokines and molecules of the arachidonic acid pathway are generated and released by microglia. The monoamine norepinephrine reduces the production of cytokines by activated microglia *in vitro*. However, little is known about the effects of norepinephrine on prostanoid synthesis. In the present study, we investigate the role of norepinephrine on cyclooxygenase- (COX-)2 expression/synthesis and prostaglandin (PG)E_2 _production in rat primary microglia.

**Results:**

Interestingly, norepinephrine increased COX-2 mRNA, but not protein expression. Norepinephrine strongly enhanced COX-2 expression and PGE_2 _production induced by lipopolysaccharide (LPS). This effect is likely to be mediated by β-adrenoreceptors, since β-, but not α-adrenoreceptor agonists produced similar results. Furthermore, β-adrenoreceptor antagonists blocked the enhancement of COX-2 levels induced by norepinephrine and β-adrenoreceptor agonists.

**Conclusions:**

Considering that PGE_2 _displays different roles in neuroinflammatory and neurodegenerative disorders, norepinephrine may play an important function in the modulation of these processes in pathophysiological conditions.

## Background

Microglia, the innate immune cells of the brain, constantly screen their microenvironment and transform into an "activated" state in response to brain lesions, e.g., toxic lesions or debris and degenerating neurons [1,2] (for review see [3]). Once activated, microglia secrete pro- and anti-inflammatory mediators such as cytokines and prostaglandins (for review see [4]). *In vitro *stimulation with lipopolysaccharide (LPS), the endotoxin of gram-negative bacteria, results in the secretion of neurotoxic and pro-inflammatory mediators. LPS triggers the activation of microglial cells via the anchored surface myeloid glycoprotein CD14 [5]. CD14 has also been found to bind to amyloid-β (Aβ), the major compound found in amyloid plaques in the brains of patients with Alzheimer's disease (AD) [6].

LPS is known to induce the production of cyclooxygenase-2 (COX-2) enzyme in microglial cells *in vitro *[7-9]. COX-2 converts arachidonic acid released from membrane phospholipids to prostaglandin (PG) H_2_. PGH_2 _is then isomerized to PGE_2 _by terminal prostaglandin E synthases. COX-2 has emerged as a major player in inflammatory reactions in the brain and increased COX-2 expression has been considered to contribute to neurodegeneration [10,11]. Elevated COX-2 expression has been described in AD [12-17] and COX-2 protein content in the hippocampus of AD patients may correlate with the severity of dementia [18] On the other hand, COX-2 has been suggested to play a physiological role in the brain for being involved in neuronal plasticity and synaptic transmission [19,20].

In recent years it has become evident that there may exist a crosstalk between the autonomic nervous system and the immune system during inflammation [21,22]. The catecholamine norepinephrine (NE) is a classical neurotransmitter with suggested immunomodulatory properties. NE is released by neurons into the synaptic cleft and may exert effects on glial cells that are in close vicinity. NE binds to α- and β-adrenergic receptors, and expression of α_1_-, α_2_-, β_1_- and β_2_-adrenegic receptors have been identified on microglia [23-29].

An immunosuppressive role has been suggested for NE *in vitro *since it attenuates LPS-induced microglial production of tumour necrosis factor (TNF)α, interleukin (IL)-1β, IL-6 and nitric oxide *in vitro *[24,30,31]. NE has been shown to reduce microglia-induced neuronal cell death [30,32].

Although it is known that NE has immunosuppressive properties, information of NE's effect on prostaglandins is scarce. The aim of our study was to further investigate the role of NE on eicosanoid production, namely PGE_2_, in primary rat microglia.

## Methods

### Chemicals and reagents

L-(-)-Norepinephrine (+) bitartrate salt monohydrate (A9512), LPS, the α_1_-adrenergic agonist L-phenylephrine hydrochloride and the β_2_-adrenergic agonist terbutaline hemisulfate salt were obtained from Sigma-Aldrich (Taufkirchen, Germany). ICI 118,551, a selective β_2_-antagonist, and CGP 20712A, a selective β_1_-antagonist, the α_2_-receptor agonist clonidine, the α_1_-receptor antagonist nicergoline, and the β_1_-/β_2_-receptor agonist dobutamine were obtained from Tocris (distributed by Biotrend, Cologne, Germany).

### Cell culture

Primary microglial cell cultures were established from cerebral cortices of one-day-old neonatal Wistar rats as previously described [7,33]. Briefly, forebrains were minced and gently dissociated by repeated pipetting in Hank's balanced salt solution. Cells were collected by centrifugation, resuspended in Dulbecco's modified Eagle's medium containing 10% fetal calf serum and antibiotics and cultured on 10-cm cell culture dishes (Falcon, 5 × 10^5 ^cells/plate) in 5% CO_2 _at 37°C. Medium was prepared taking extreme care to avoid LPS contamination [34]. Floating microglia were harvested from 10- to 14-day-old mixed (astrocyte-microglia) primary cultures and re-seeded into 35-mm cell culture dishes in fresh complete medium to give pure microglial cultures (2 × 10^4 ^cells/dish). Microglial cultures were washed 1 h after seeding to remove non-adherent cells. The purity of the microglial culture was >98% as previously determined by immunofluorescence and cytochemical analysis [34].

### RNA extraction and RT-PCR analysis

Total RNA was isolated using the guanidine isothiocyanate method [35]. Two μg of total RNA was reverse transcribed using M-MLV reverse transcriptase and random hexamers (Promega, Mannheim, Germany). One μl of the resulting cDNA was amplified using Taq DNA polymerase (Promega), dNTPs (Invitek, Berlin, Germany) and primers specific for rat COX-1 (forward, 5'-CGG CCT CGA CCA CTA CCA ATG-3'; reverse, 5'-TGC GGG GCG GGA ATG AAC T-3', annealing temperature 60°C, 30 cycles, amplicon size: 426 bp); rat COX-2 (forward: 5' TGC GAT GCT CTT CCG AGC TGT GCT 3', reverse: 5' TCA GGA AGT TCC TTA TTT CCT TTC 3', annealing temperature 55°C, 35 cycles, amplicon size: 479 bp); rat S12 (forward: 5'-ACG TCA ACA CTG CTC TAC A-3', reverse: 5'-CTT TGC CAT AGT CCT TAA C-3', 56°C, 30 cycles, amplicon size: 312 bp), that were designed using Primer Select software (DNA Star Inc., Madison, WI) and synthesized through an in-house facility (Dr. Gabor Igloi, Institute for Biology III, Freiburg, Germany). All PCR amplifications included a final 10-min extension at 72°C. The products were analyzed on a 2% agarose gel. Contamination by genomic DNA was identified by substituting total RNA instead of cDNA in the reaction mixture using S12 primers.

### Western blot analysis

After the respective experimental set up, microglial cells were washed with phosphate-buffered saline (PBS) and lysed in 1.3 × SDS- (sodium dodecyl sulfate-) containing sample buffer without DTT or bromophenol blue containing 100 μM orthovanadate [36]. Lysates were homogenized by repeated passage through a 26-gauge needle. Protein contents were measured using the bicinchoninic acid (BCA) method (kit obtained from Pierce, distributed by KFC Chemikalien, München, Germany). Bovine serum albumin (BSA) was used as a protein standard at concentrations ranging from 0.2 μg/μl to 4 μg/μl; the optical density was read at 570 nm using a microplate reader. Before electrophoresis, bromophenol blue and DTT (final concentration, 10 mM) were added to the samples. For COX-1 and COX-2 immunoblotting, 30 to 50 μg of protein from each sample was subjected to SDS-PAGE (polyacrylamide gel electrophoresis) on a 10% gel under reducing conditions. Proteins were then transferred onto a polyvinylidene fluoride (PVDF) membrane (Millipore, Bedford, MA, USA) by semi-dry blotting. The membrane was blocked for 1 or 2 h at room temperature using Rotiblock (Roth, Karlsruhe, Germany) or 5% blocking milk (BioRad, München, Germany), before the overnight incubation at 4°C with the primary antibody. Primary antibodies were goat anti-COX-1 goat (M-20, Santa Cruz, Heidelberg, Germany) and anti-COX-2 diluted 1:500 in Tris-buffered saline (TBS) containing 0.1% Tween 20 (Merck, Darmstadt, Germany) and 1% bovine serum albumin (BSA) and rabbit anti-actin diluted 1:5000 (Sigma, St. Louis, MO, USA). After extensive washing (three times for 15 min each in TBS containing 0.1% Tween 20), proteins were detected with horseradish peroxidase (HRP)-coupled rabbit anti-goat IgG (Santa Cruz Biotechnology Inc., Santa Cruz, CA, USA, 1:100,000) or HRP-coupled donkey anti-rabbit (GE Healthcare, Freiburg Germany, 1:25,000) using chemiluminescence (ECL) reagents (GE Healthcare). All western blot experiments were carried out at least three times.

### Enzyme immunoassay (EIA)

Supernatants were harvested, centrifuged at 10,000 × g for 10 min, and levels of prostaglandin E_2 _in the media were measured by enzyme immunoassay (EIA) (Assay design, distributed by Biotrend, Cologne, Germany and Cayman Chemicals, Ann Arbor, MI, USA, respectively) according to the manufacturer's instructions. Standards from 39 to 2500 pg/ml were used; sensitivity of the assay was 36.2 pg/ml.

### Statistical analysis

At least three independent experiments were used for data analysis. Original data were converted into %-values of LPS control and mean ± S.E.M. were calculated. Values were compared using *t*-test (two groups) or one-way ANOVA with *post-hoc *Student-Newman-Keuls test (multiple comparisons). Differences were considered statistically significant when p < 0.05.

## Results

### NE enhances LPS-induced production of COX-2 mRNA and protein in primary rat microglial cells

**Figure 1:**
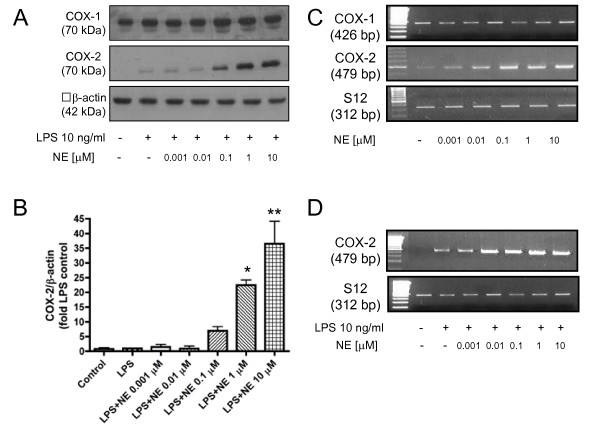
**Different concentrations of norepinephrine enhance the synthesis of COX-2 protein (A, B) and mRNA (C) in LPS-stimulated primary neonatal microglial cells**. Microglial cells were stimulated with different concentrations of norepinephrine (0.001 μM to 10 μM) and LPS (10 ng/ml) for 4 hours. (A) A representative western blot against β-actin (42 kDa) and COX-2 (70 kDa) is shown here. (B) Quantitative densitometric analysis of COX-2 protein expression normalized to β-actin control (n = 3). *P < 0.05, **P < 0.001 with respect to LPS control. (C, D) Semi-quantitative PCR analysis of the effect of different concentrations of norepinephrine alone (C) and of norepinephrine plus LPS on COX-2 and COX-1 mRNA expression (D). [NE = norepinephrine].

We show here that NE dose-dependently enhanced LPS-induced COX-2 protein levels in primary rat microglia (Fig. 1A). No COX-2 protein levels were detected after 4 h stimulation with LPS (10 ng/ml) alone. Moreover, NE alone showed no effect. Addition of 1-100 nM NE to LPS-stimulated cells did not increase COX-2 protein levels, although 100 nM NE in combination with LPS already showed a tendency to increase COX-2 levels. Significant upregulation of COX-2 immunoreactivity was observed at doses of 1 to 10 μM NE in combination with LPS. Analysis of western blots from three independent experiments showed a 22- and 35-fold increase in COX-2 immunoreactivity in rat primary microglial cells (p < 0.001) using 1 and 10 μM NE, respectively, as compared to 10 ng/ml LPS alone (Fig. 1B). On the other hand, we did not observe any alterations in COX-1 protein levels after adding the same doses of NE along with LPS (Fig. 1A).

Next, we investigated whether our findings on the protein level were due to increased mRNA levels, using qualitative PCR. COX-2 mRNA was marginally detectable in unstimulated microglial cells. In contrast to the finding that NE alone did not influence COX-2 protein levels, a potent induction of COX-2 mRNA expression was observed after 2 h stimulation with NE alone, which resulted in a dose-response upregulation starting with 1 nM that peaked at 10 μM NE (Fig. 1C). Addition of LPS, 10 ng/ml, further enhanced the expression of COX-2 mRNA (Fig. 1D). Thus, in agreement with the effects observed on COX-2 protein levels, we found an increase in COX-2 mRNA when microglial cells were stimulated by both LPS plus NE.

**Figure 2:**
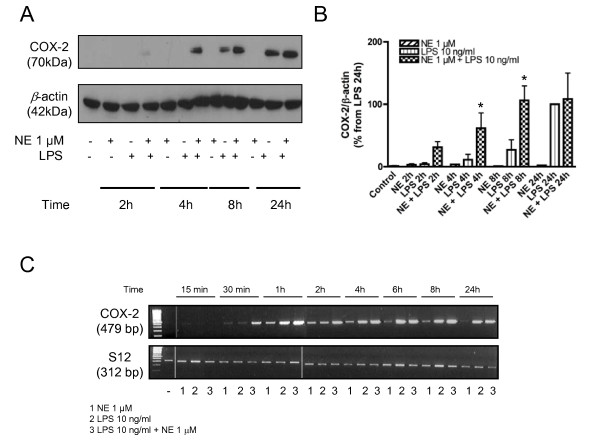
**Time course of treatment with norepinephrine 1 μM alone, LPS 10 ng/ml alone, or a combination of norepinephrine 1 μM plus LPS 10 ng/ml on the expression of COX-2 protein and mRNA (A-C)**. (A, B) Microglial cells were stimulated for 4, 8, and 24 hours. This was followed by western blot analysis against β-actin (42 kDa) and COX-2 (70 kDa). A representative western blot is shown here (A). Quantitative densitometric analysis of COX-2 normalized to β-actin loading control. *P < 0.05 with respect to LPS control. (C) Semi-quantitative analysis of the effect of the different treatment groups on COX-2 mRNA expression at different time points (15 min to 24 hours). [NE = norepinephrine].

Next we studied time kinetics in COX-2 mRNA and protein synthesis after stimulation with 10 ng/ml LPS or 1 μM NE and the combination of both. At 2 h, a slight increase in COX-2 immunoreactivity was detected when microglia were treated with LPS plus NE, while there was no change in COX-2 protein levels when the cells were treated with LPS or NE alone (Fig. 2A). LPS plus NE further increased COX-2 immunoreactivity significantly at 4 and 8 h compared to LPS treatment alone (p < 0.05). After 24 h, LPS alone reached the same immunoreactivity as the treatment of LPS plus NE (Fig. 2A and 2B).

COX-2 mRNA upregulation was already observed at 30 min upon LPS plus NE treatment and reached its maximum by 1 h. In LPS-only treated microglial cells COX-2 mRNA expression levels reached a maximum and the same expression level as LPS plus NE after 6 h. In LPS only and LPS plus NE, COX-2 level did not further increase and stayed at the same level after 24 h. Interestingly, NE alone also led to an increase in COX-2 mRNA expression by 30 min and was still observed after 8 h (Fig. 2C).

### NE increases release of PGE2 by LPS-stimulated primary microglial cells

**Figure 3:**
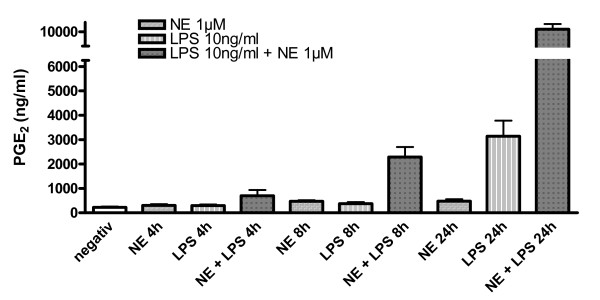
**Effect of norepinephrine on PGE_2 _concentrations at different time points (0 h, 4 h, 8 h, 24 h) was measured in the supernatants of LPS-stimulated primary microglial cell cultures using EIA (n = 6)**. Data are depicted as means ± standard error of mean. Statistical analysis was done using ANOVA with post hoc Newman-Keuls for the 4 different time points (*P < 0.001). [NE = norepinephrine].

We further investigated whether the augmentation of COX-2 synthesis by NE in the presence of LPS is accompanied by an increased release of PGE_2_, an important product of the enzymatic activity of COX-2 (Fig. 3). PGE_2 _was barely detectable in unstimulated primary neonatal microglial cells. Treatment with NE, 1 μM alone, did not lead to an increase in PGE_2 _over 24 h. LPS, 10 ng/ml alone, significantly increased PGE_2 _concentration after 24 h (p < 0.05), whereas there was no significant increase after 4 and 8 h stimulation with LPS.

We observed a significant increase in the secretion of PGE_2 _after 8 h of stimulation with the combination of LPS (10 ng/ml) and NE (1 μM) compared to unstimulated cells (p < 0.05). At 24 h, LPS plus NE also showed a significant elevation of PGE_2 _levels compared to treatment with LPS alone (p < 0.001). The enhancement of PGE_2 _induced by NE in LPS-stimulated microglia, but not by NE alone, confirms the previous results in which NE in combination with LPS further increased COX-2 immunoreactivity whereas NE alone did not increase COX-2 protein levels.

### The NE-induced increase in COX-2 is mediated by β-adrenoreceptors

**Figure 4:**
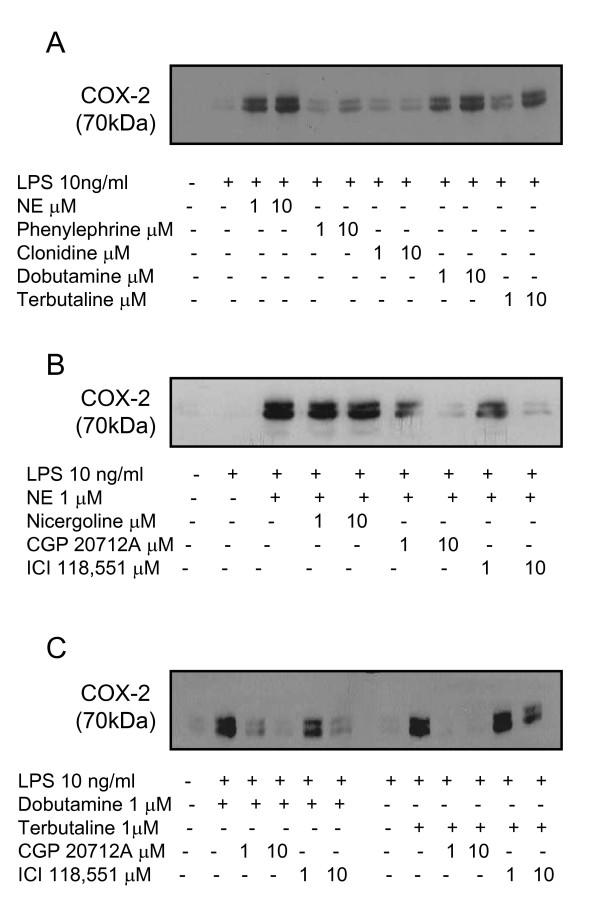
**Effect of different α- and β-adrenergic agonists and antagonists on COX-2 protein expression**. Microglial cells were stimulated with LPS 10 ng/ml and different adrenergic agonists/antagonists for 4 hours, followed by western blot analysis for COX-2 protein expression (70 kDa). (A) LPS-stimulated microglial cells were treated with different α- and β-adrenoreceptor agonists at two different concentrations (1 and 10 μM): α_1_-agonist phenylephrine; α_2_-agonist clonidine; β_1_/β_2_-agonist dobutamine; β_2_-agonist terbutaline. (B) LPS-stimulated microglial cells were treated with different α- and β-adrenoreceptor antagonists at two different concentrations (1 and 10 μM): α_1_/α_2_-antagonist nicergoline; β_1_-antagonist CGP 20712A; β_2_-antagonist ICI118, 551. (C) LPS-stimulated microglial cells were treated both with dobutamine or terbutaline and with the two different β-adrenoreceptor antagonists ICI 118,551 or CGP 20712A. [NE = norepinephrine].

Since microglial cells express α- and β-adrenoreceptors, and NE activates G-protein-coupled receptors, we asked which adrenoreceptor subtype mediates the increase in COX-2 after stimulation with LPS in combination with NE. Thus, we treated microglial cells with LPS, 10 ng/ml, in combination with various adrenoreceptor agonists (Fig. 4A). Co-treatment of LPS with the selective α_1_-agonists phenylephrine only induced a modest increase in COX-2 protein expression compared to LPS and NE treatment, while clonidine, an α_2_-adrenergic agonist, did not show any effect at all. In contrast, terbutaline and dobutamine, β_2_-and unselective β-adrenergic agonists, respectively, mimicked the effect of NE (Fig. 4A). Thus, stimulation of microglial β_1_- or β_2_-receptors, or both, seem to be required for the observed effect on COX-2 protein levels, suggesting that treatment with β-adrenergic antagonists should abolish the enhanced COX-2 protein synthesis. Indeed, the selective β_1_- and β_2_-receptor antagonists CGP 20712A and ICI 118,551, respectively, reduced COX-2 protein levels induced by the combination of LPS/NE (Fig. 4B). However, they were not able to completely abolish COX-2 immunoreactivity (Fig. 4B). Nicergoline, an α_1_/α_2_-adrenoreceptor antagonist, had no effect on COX-2 protein levels induced by NE/LPS, further supporting the absence of role of α_1_/α_2_-adrenoreceptors.

Finally, we treated LPS-stimulated microglial cells with both β-adrenergic receptor agonists and antagonists (Fig. 4C). As shown, ICI 118,551 and CGP 20712A reduced COX-2 expression in LPS- plus dobutamine-stimulated cells. After stimulation with the β_2_-selective agonist terbutaline plus LPS, addition of the β_1_-receptor antagonist CGP completely abolished COX-2 expression. Low concentrations of ICI 118,551, however, did not alter COX-2 expression levels, while at higher concentrations a modest decrease was observed.

## Discussion

In the present study, basal COX-2 expression in non-stimulated rat primary neonatal microglia was not detectable, suggesting that either COX-2 is generated by de novo synthesis in response to applied stimuli or, alternatively, that basal levels do not reach the threshold of immunoblot detection. NE alone induced COX-2 mRNA expression but did not affect COX-2 protein synthesis. We suppose that the COX-2 mRNA induced by NE is degraded before protein synthesis is initiated. Alternatively, a second stimulus, such as LPS, is required to set off translation of COX-2.

Different kinases are important in the regulation of COX-2 in LPS-stimulated microglia [37], and it has been demonstrated that NE increases the activity of mitogen-activated protein kinases (MAPK) [38] as well as transcription factors [39]. Kan et al. (1999) demonstrated that, in neonatal rat cardiac myocytes, NE alone increases the activity of MAPK and, although IL-1β alone does not induce the same effect, the co-addition of IL-1β and NE results in an enhanced MAPK activity in comparison to the substances alone [38]. Salmeterol and isoproterenol, β2- and non-selective β-adrenergic receptor agonists, respectively, enhance the phosphorylation of p38 MAPK and extracellular signal-regulated kinases (ERK) in peritoneal macrophages and RAW264.7 [40,41]. Interestingly, pretreatment with isoproterenol decreases the release of TNFα, IL-12 and NO in LPS-stimulated macrophages. On the contrary, isoproterenol reduces the release of these same mediators after PMA stimulation, indicating that the effect of isoproterenol might depend on the stimulus [42].

Recently, Morioka et al. (2009) demonstrated that, in rat spinal microglia, NE reduces phosphorylation of p38 MAPK, induced by ATP, via β_1_- and β_2_-receptors [43]. The deactivation of p38 would lead to a decrease in COX-2 protein synthesis, since p38 is involved in COX-2 mRNA stabilization [44]. As such, it is possible that the observed increase in COX-2 mRNA may be due to increased transcription or increased stability of mRNA in rat microglia by activation of certain transcription factors and/or kinases. That could explain the increase in the COX-2 mRNA with incubation of cells with NE alone, or the enhancement of COX-2 protein synthesis when associated with LPS.

On the other hand, many post-transcriptional factors contribute to the translation of mRNA, which might not be affected by NE. This could explain the lack of effect of NE on COX-2 protein synthesis. For example, it is known that LPS induces the activation of the mammalian target of rapamycin (mTOR) [45]. Activation of mTOR induces translation of different mRNA through its downstream targets such as the ribosomal p70S6 kinase and the initiation factor 4E-binding protein 1 [46]. Although we did not test this possibility, it seems feasible that NE *per se *cannot activate the machinery responsible for the translation of COX-2 in rat microglia, but potentiates protein synthesis by increasing transcription or protein stability.

As shown before, LPS, 10 ng/ml, increases COX-2 mRNA and protein expression at 4 and 8 h, respectively [8]. Co-stimulation with NE already at low concentrations results in earlier and a markedly enhanced induction of COX-2 mRNA and protein levels. Similar to our results, other groups have also shown that NE *per se *does not increase protein synthesis, but drastically increases the effect induced by LPS. In peripheral blood monocytes and monocyte-derived macrophages, NE and epinephrine alone only show a minor effect on matrix metalloproteinase (MMP)-1 and MMP-9 production [39]. However, a combination of the catecholamine with LPS further enhances the increased production of MMP-1 and MMP-9.

Next, we investigated whether the increased intracellular levels of COX-2 evoked by LPS plus NE causes elevated levels of PGE_2_. In our experiments, NE plus LPS further increased the levels of PGE_2_. Interestingly, after 24 h stimulation with NE plus LPS, COX-2 mRNA and protein levels are similar to the increase observed in LPS alone. However, the increase observed in PGE_2 _is about three-fold higher in LPS plus NE than with LPS alone. Thus, it is possible that the combination of LPS and NE might induce the expression or increase the activity of other enzymes involved in PGE_2 _synthesis, such as phospholipase A_2 _and/or PGE synthases.

Dependent on the experimental setting, PGE_2 _can exert either neuroprotective or neurodetrimental effects. Agents reducing PGE_2 _synthesis in *in vitro *and *in vivo *models of chronic neurodegenerative diseases like Parkinson's disease or AD have been demonstrated to be neuroprotective due to their anti-inflammatory effects [47-52]. On the other hand, exogenous PGE_2 _protects neurons from LPS-induced cell death by reduction of NO and reactive oxygen species [53]. Direct administration of PGE_2 _into the brain has also been shown to reduce microglial activation and TNF-α expression in brain parenchyma induced by intraperitoneal LPS injection [54]. In addition, PGE_2 _protects neurons in culture from different types of noxious stimuli [29,55,56].

Localized inflammatory responses in the brain parenchyma have been associated with the pathogenesis and progression of AD. Inhibition of neuroinflammation has been identified as a potential therapeutic target [57-60]. COX-2 expression is elevated in the AD brains [14,18] and PGE_2 _is accumulated in the cerebrospinal fluid of AD patients [61]. It was therefore reasonable to hypothesize that inhibition of COX-2 may have a therapeutic potential in AD. Despite convincing evidence in epidemiological studies on the prevention of AD through long-term treatment with non-steroidal anti-inflammatory drugs, most clinical trials have failed to show beneficial effects [62-65], suggesting that either the molecular target or the therapeutic window has been missed.

PGE_2 _acts on four different receptors, EP1-EP4 [66], of which microglia express three, namely EP1, EP2, and EP3; the latter having been exclusively detected in activated microglia [67]. Microglial EP2 receptors are known to enhance neurotoxic activities [10,50-52,68]. This would suggest that enhanced secretion of PGE_2 _through NE might increase microglial toxicity. However, the role of PGE_2 _may be far more complex due to the presence of other receptor subtypes on microglia. So far, NE-mediated regulation of EP receptor expression on microglia has not been studied.

In this study, we could show that the observed effect of NE is mediated by β-adrenoreceptor agonists. This corresponds to the findings of Minghetti and Levi, who showed that the non-selective β-adrenergic agonist isoproterenol increases COX-2 protein and PGE_2 _synthesis in microglial cells [8]. In our experiments, both β_1_- and β_2_-receptors seem to mediate the enhanced effect of COX-2 production. Our data also indicate a lack of involvement of α-adrenoreceptors. The use of relatively-selective β-adrenoreceptors allowed us to further confirm the participation of β-adrenoreceptors in the enhancement of LPS-induced COX-2 expression. Based on our data, both β-adrenoreceptors subtypes seem to be involved. The antagonists used in this study, CGP20712A (β_1_-antagonist) and ICI118,551 (β_2_-antagonist) are widely used to discern the role of β-adrenoreceptor subtypes [69,70]. Terbutaline is considered a relatively selective β_2_-agonist, but it also binds to the β_1_-adrenoreceptor [71]. As with any pharmacological agent, these compounds are not 100% selective, therefore future studies utilizing knockout animals will be needed to fully clarify the role of distinct β-adrenoreceptor subtypes.

We did not detect that stimulation of α-adrenoreceptors results in any significant increase in COX-2 protein levels. Of note, stimulation of β-adrenoreceptors increases levels of cAMP in microglial cells, mainly through activation of the β_2_-adrenoreceptor [25]. Raised intracellular cAMP levels are known to suppress activation of microglial cells [54,72,73]. Increased PGE_2 _levels may in addition stimulate the microglial EP2 receptor, which is linked to cAMP formation [74] and thereby contribute to the inactivation of microglia. Other cell culture experiments have shown that exogenous PGE_2 _results in decreased levels of pro-inflammatory cytokines such as TNF-alpha and IL-12 in LPS-stimulated microglia [75-77]. In concordance with these results, increased cAMP levels via stimulation of β-adrenoreceptors inhibits the production of macrophage inflammatory protein (MIP)-1α, which is known to activate macrophages to secrete pro-inflammatory cytokines [78].

Our data suggest that NE has a strong effect on microglial inflammatory responses, suggesting that NE is an active modulator of microglial activation. This may be important in AD, a disease in which early loss of noradrenergic locus coeruleus (LC) neurons has been observed [79]. Depletion of LC neurons by injection of the neurotoxin DSP4 increases the levels of inflammatory mediators like iNOS, IL-1β and IL-6 [80].

## Conclusions

Our study shows that NE increases LPS-induced COX-2 expression and PGE_2 _secretion by primary rat neonatal microglia *in vitro*. These results shed new light on the role of NE in CNS inflammation. NE via COX-2 and PGE-2 induction may have a protective physiological function rather than being purely detrimental. However, further investigations in *in vivo *models, especially in regard to PGE_2 _and COX-2, may help to clarify the exact role of NE in neuroinflammation.

## Competing interests

The authors declare that they have no competing interests.

## Authors' contributions

JCMS contributed to the design of the study, carried out western blot analysis, performed the statistical analysis and wrote the manuscript; BLF directed the work of the study, reviewed the data and the manuscript; EH participated in western blot analysis and prostaglandin measurements; ECJ and ACPO provided consultation and reviewed the manuscript; MTH was involved in drafting the manuscript and revised it critically; MH conceived of the study, directed the work and reviewed the manuscript. All authors read and approved the final manuscript.
